# Kinase and Phosphatase Cross-Talk at the Kinetochore

**DOI:** 10.3389/fcell.2018.00062

**Published:** 2018-06-19

**Authors:** Adrian T. Saurin

**Affiliations:** Jacqui Wood Cancer Centre, School of Medicine, Ninewells Hospital and Medical School, University of Dundee, Dundee, United Kingdom

**Keywords:** kinase, phosphatase, spindle assembly checkpoint, kinetochore, Cdk1, PP2A, PP1, mitosis

## Abstract

Multiple kinases and phosphatases act on the kinetochore to control chromosome segregation: Aurora B, Mps1, Bub1, Plk1, Cdk1, PP1, and PP2A-B56, have all been shown to regulate both kinetochore-microtubule attachments and the spindle assembly checkpoint. Given that so many kinases and phosphatases converge onto two key mitotic processes, it is perhaps not surprising to learn that they are, quite literally, entangled in cross-talk. Inhibition of any one of these enzymes produces secondary effects on all the others, which results in a complicated picture that is very difficult to interpret. This review aims to clarify this picture by first collating the direct effects of each enzyme into one overarching schematic of regulation at the Knl1/Mis12/Ndc80 (KMN) network (a major signaling hub at the outer kinetochore). This schematic will then be used to discuss the implications of the cross-talk that connects these enzymes; both in terms of why it may be needed to produce the right type of kinetochore signals and why it nevertheless complicates our interpretations about which enzymes control what processes. Finally, some general experimental approaches will be discussed that could help to characterize kinetochore signaling by dissociating the direct from indirect effect of kinase or phosphatase inhibition *in vivo*. Together, this review should provide a framework to help understand how a network of kinases and phosphatases cooperate to regulate two key mitotic processes.

## Introduction

The kinetochore is a molecular complex of at least 100 different proteins that assembles on the centromeric region of chromosomes to allow their attachment to microtubules during mitosis (Cheeseman, 2014; Nagpal and Fukagawa, 2016; Pesenti et al., 2016; Musacchio and Desai, 2017). As well as providing a structural platform for microtubules to bind, the kinetochore also safeguards this attachment process in two ways: (1) it activates the spindle assembly checkpoint (SAC) to arrest cells in mitosis until all sister kinetochores have attached to microtubules emanating from opposite spindle poles (termed bi-orientation) (Musacchio, 2015; Joglekar, 2016; Corbett, 2017). (2) It ensures this bi-orientation occurs correctly by sensing and destabilizing incorrect attachments that do not generate sufficient tension, in a process known as error-correction (Cheerambathur and Desai, 2014; Sarangapani and Asbury, 2014; Krenn and Musacchio, 2015; Lampson and Grishchuk, 2017). Both of these processes are regulated at the KMN network, which acts as a platform for microtubule attachment and SAC signaling at the outer kinetochore (Varma and Salmon, 2012; Foley and Kapoor, 2013).

The KMN network is composed of 10 different proteins that map to three separate subcomplexes: the Knl1 complex (containing Knl1 and Zwint), the Mis12 complex (containing Mis12, Pmf1, Dsn1, Nsl1), and the Ndc80 complex (containing Ndc80, Nuf2, Spc24, and Spc25) (Musacchio and Desai, 2017). The Mis12 complex acts to structurally tether the KMN network to chromatin by binding to the constitutive centromere-associated network (CCAN). The Ndc80 and Knl1 complexes, on the other hand, are the key regulatory hubs that bind to microtubules and scaffold SAC signaling, respectively (Caldas and Deluca, 2014; Cheerambathur and Desai, 2014; Musacchio and Desai, 2017).

Although the SAC and microtubule attachment processes are very distinct, they must be precisely coordinated in time and space. For example, as soon as a kinetochore makes a correct end-on attachment to microtubules, local SAC signaling must be rapidly extinguished. It is perhaps not surprising, therefore, that at a molecular level these processes are extremely well connected. In fact, they are each regulated by an overlapping network of enzymes that includes at least five kinases (Aurora B, Mps1, Bub1, Plk1, and Cdk1) and two phosphatases (PP1 and PP2A-B56) (Funabiki and Wynne, 2013; Vallardi et al., 2017). Although these enzymes undoubtedly have very specific roles at the kinetochore, sometimes in only one particular process, their multiple interconnections mean that it is incredibly difficult to dissociate their direct from indirect effects.

**Figure 1:**
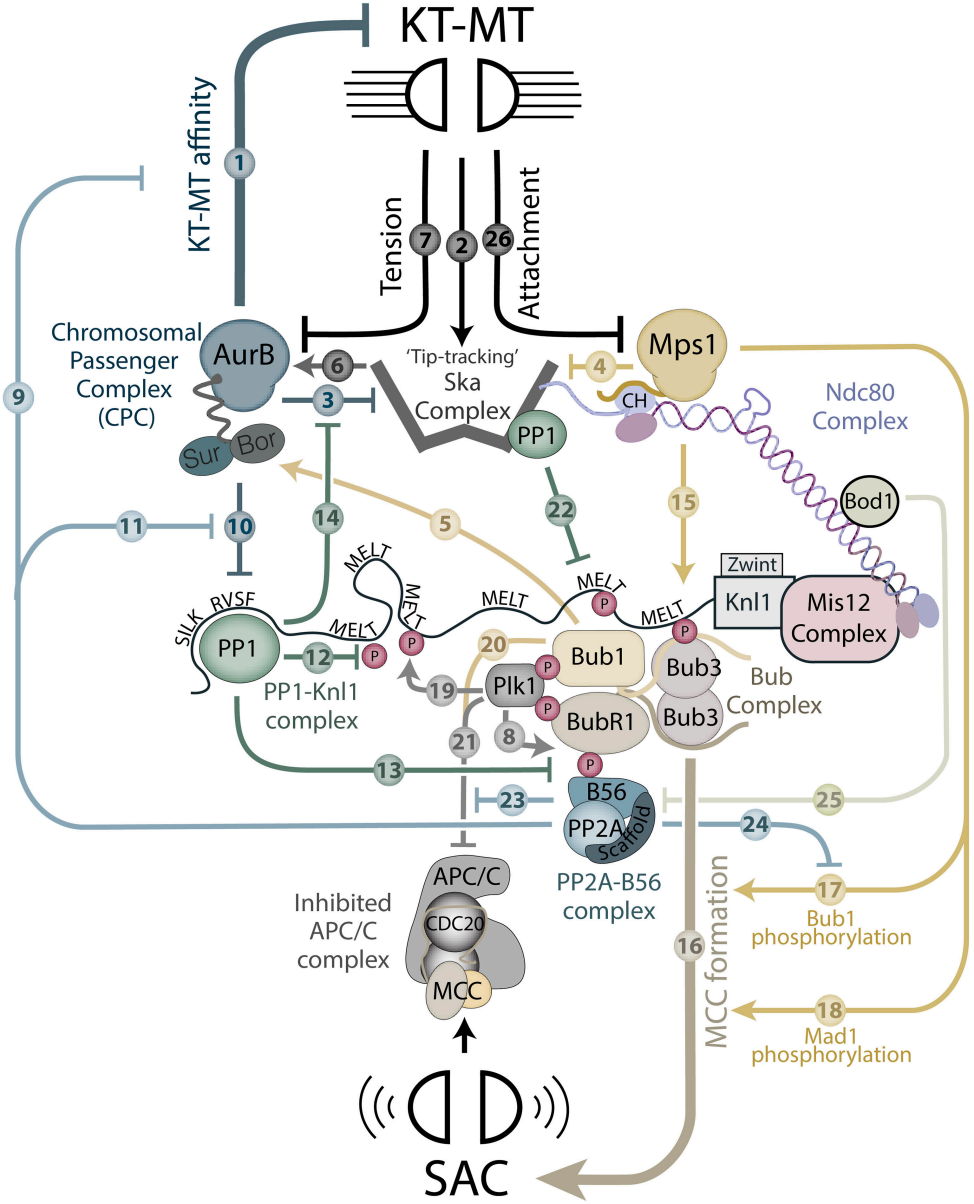
A schematic to depict kinase and phosphatase regulation at the KMN network. The model displays regulatory components that are known to localize to the KMN network and regulate either kinetochore-microtubule (KT-MT) attachments or the SAC. The regulatory inputs are indicated by numbered arrows that are explained fully at the appropriate place in the text.

In this review I will attempt to untangle this complicated picture by first summarizing the established direct effects of each particular enzyme at the KMN network. These connections are depicted in Figure 1, which should be referenced in conjunction with the text below where each of the arrows will be mechanistically explained (the arrows are numbered and labeled in the text to highlight where they are discussed). Even before I delve into the mechanistic details, however, a quick glance at this network will already reveal how interfering with any one of these enzymes can produce knock-on effects for all the others. The result is that if either one is inhibited specifically, there will be consequential effects for both kinetochore-microtubule attachments and the SAC. Therefore, the question of exactly “who controls what” becomes a very difficult one to answer definitively, which has most likely contributed to confusion and controversy within the field. To help to resolve these issues, after fully explaining Figure 1, I will then focus on each enzyme individually to discuss some past and present questions relating to the issue of direct vs. indirect effects. Finally, I will highlight some general experimental approaches that could be used in future to address some of the issues that still remain to be resolved.

## Kinetochore-microtubule attachment and error-correction

During mitosis, kinetochore pairs must become bioriented on the mitotic spindle (i.e., one kinetochore attached to one spindle pole and its sister kinetochore attached to the other). If this configuration is achieved for every single kinetochore pair, then upon mitotic exit, the sister chromatids will be split in opposing directions and each daughter cell will receive the correct complement of the genome. Since kinetochore-microtubule interactions form stochastically, this whole process must be regulated to ensure that improper attachments are continually removed, in a process termed error-correction (Cheerambathur and Desai, 2014; Lampson and Grishchuk, 2017).

A fundamental principle of error-correction is that incorrectly attached microtubules are detached by the phosphorylation of key binding interfaces at the kinetochore, many of which exist on the KMN network (Cheeseman, 2014). Importantly, as soon as the correct type of tension-generating attachments are formed, these interfaces are dephosphorylated (or remain in the unphosphorylated state), and the force-producing microtubule attachments are rapidly stabilized. Tension itself plays a major role in error-correction, as kinetochores appear able to “sense” the level of tension and only phosphorylate and detach microtubules when this tension state is low. Many other factors, including spindle geometry and microtubule tip dynamics, also contribute to error-correction, and the reader is directed toward some excellent recent reviews to learn about this process in more detail (Sarangapani and Asbury, 2014; Lampson and Grishchuk, 2017). Here, I will only present the key facts that are important to understand how enzymes at the KMN network can regulate the attachment of microtubules to the kinetochore.

Aurora B kinase phosphorylates numerous substrates at the kinetochore to inhibit microtubule binding (Krenn and Musacchio, 2015). The best characterized of these is Ndc80, which is phosphorylated by Aurora B on multiple residues in its N-terminal tail to detach microtubules (Cheeseman et al., 2006; Deluca et al., 2006; Wei et al., 2007; Ciferri et al., 2008; Guimaraes et al., 2008; Miller et al., 2008; Alushin et al., 2010; Zaytsev et al., 2014, 2015). In addition, Aurora B phosphorylates other kinetochore targets (Welburn et al., 2010; Hua et al., 2011), microtubule binding proteins (Iimori et al., 2016), and microtubule-depolymerizing kinesins (Andrews et al., 2004; Lan et al., 2004; Ohi et al., 2004; Zhang et al., 2007; Knowlton et al., 2009) to regulate kinetochore-microtubule affinity and/or dynamics. Collectively, these inhibitory Aurora B phosphorylations are represented by a thick inhibitory arrow from Aurora B to kinetochore-microtubule attachment in Figure 1 (arrow 1).

As well as binding to microtubules, the kinetochores must also be able to hold onto the microtubule tips as they polymerize and depolymerize. This “tip-tracking” is controlled by one of two distinct but functionally homologous complexes (depending on species; see Van Hooff et al., 2017): the DAM1 complex (found in fungi; Westermann et al., 2005, 2006; Asbury et al., 2006; Tanaka et al., 2007) and the Ska complex (found in mammals; Hanisch et al., 2006; Gaitanos et al., 2009; Raaijmakers et al., 2009; Theis et al., 2009; Welburn et al., 2009; Schmidt et al., 2012), which both bind to Ndc80 and are recruited to kinetochores by microtubules (activating arrow 2 from KT-MT in Figure 1). Importantly, Aurora B can phosphorylate and inhibit both of these complexes to detach kinetochore-microtubule fibers (Cheeseman et al., 2002; Shang et al., 2003; Gestaut et al., 2008; Lampert et al., 2010; Tien et al., 2010; Chan et al., 2012; Schmidt et al., 2012) (inhibitory arrow 3 from Aurora B in Figure 1). Aurora B may be aided by other kinetochore kinases in this respect, because Mps1 can also phosphorylate the Ska complex to prevent its ability to track depolymerizing microtubules (Maciejowski et al., 2017), and perhaps also to inhibit its localization to kinetochores (Sivakumar and Gorbsky, 2017) (inhibitory arrow 4 from Mps1 in Figure 1). Furthermore, Aurora A can also regulate kinetochore-microtubule dynamics by phosphorylating Aurora B targets at the kinetochore when they come into the vicinity of spindle poles (Chmátal et al., 2015; Ye et al., 2015) or perhaps even on aligned kinetochores at metaphase (Deluca et al., 2018) (not shown in Figure 1, because it is unclear if Aurora A itself is regulated at the KMN network). In summary, various kinases can impact on microtubule attachments to the kinetochore, however, the principal regulator of these attachments is Aurora B, which can phosphorylate a variety of different substrates to disrupt kinetochore-microtubule affinity and alter microtubule dynamics.

Aurora B is the catalytic subunit of the chromosomal passenger complex (CPC), which also contains two regulatory subunits, borealin and survivin, that are tethered to Aurora B by INCENP (for inner centromere protein; Jeyaprakash et al., 2007). The CPC binds to chromatin at the beginning of mitosis and then clusters at the centromere, where it is able to auto-phosphorylate on key activating motifs in INCENP and the Aurora B catalytic domain (Bishop and Schumacher, 2002; Honda et al., 2003; Yasui et al., 2004; Sessa et al., 2005; Ruppert et al., 2018). At least some of these phosphorylation's occur in *trans* (Sessa et al., 2005; Zaytsev et al., 2016), which explains why clustering of the CPC is important for Aurora B activation (Kelly et al., 2007; Wang et al., 2011). This requirement for clustering allows multiple different feedback loops to work together to control Aurora B localization and activity at the centromere: a histone-associated kinase (Haspin) and phosphatase (PP1-RepoMan) work together with a kinetochore-bound kinase (Bub1) to phosphorylate histone tails (Histones H2A-pT120 and H3-pT3) specifically at centromeres (for an in-depth recent review see Hindriksen et al., 2017). For the purpose of this article, it is just important to note that the centromeric recruitment of Aurora B is dependent on Knl1-localized Bub1, which phosphorylates histone H2A-T120 adjacent to the kinetochore to recruit the CPC (Kawashima et al., 2007, 2010; Tsukahara et al., 2010; Yamagishi et al., 2010) (this binding is actually mediated via a protein intermediate, shugoshin, but this is represented as a single arrow 5 from Bub1 to the CPC in Figure 1). It is also important to point out that although Aurora B is activated at the centromere, it must ultimately act at the outer kinetochore to regulate microtubule attachments. A pool of active Aurora B has been detected at or near the outer kinetochore using a phospho-Aurora B (Thr232) activation loop antibody (Posch et al., 2010; Deluca et al., 2011). Although this kinetochore-proximal pool of Aurora B remains to be fully characterized, it appears to require the KNL N-terminus (Caldas et al., 2013) and CPC dimerization (Bekier et al., 2015). Identifying the relevant binding site(s) for Aurora B at the outer kinetochore remain an important future goal. At least one potential binding site is the Ska complex, which can directly bind Aurora B and enhances its catalytic activity (activating arrow 6 in Figure 1) (Redli et al., 2016).

A key aspect of error-correction is the ability of Aurora B to discriminate between different forms of kinetochore-microtubule attachments, such that only the incorrect types are destabilized. This is achieved because bipolar attachments generate sufficient tension to inhibit Aurora B activity at the kinetochore, whereas improper attachments do not (inhibitory arrow 7 from KT-MT to Aurora B in Figure 1). Exactly how tension reduces Aurora B activity is a matter of considerable debate, although it is likely to involve an increase in centromere-kinetochore distance and/or structural changes within the kinetochore itself (Cheerambathur and Desai, 2014; Sarangapani and Asbury, 2014; Krenn and Musacchio, 2015; Lampson and Grishchuk, 2017). These structural changes may impact on the activities of Aurora B, its antagonizing phosphatase(s), and/or the accessibility of Aurora B substrates at the kinetochore.

In mammalian cells, a key phosphatase that supresses Aurora B activity is PP2A-B56 (Foley et al., 2011), which localizes to the KMN network by binding to the kinetochore-attachment regulatory domain (or “KARD”) of BubR1 (Suijkerbuijk et al., 2012; Kruse et al., 2013; Xu et al., 2013; Wang J. et al., 2016): BubR1 is a KNL1-localized checkpoint protein that will be discussed in detail later. The BubR1-KARD conforms to an LxxIxE sequence that targets a variety of different substrates and adaptors to PP2A by binding to a conserved pocket on the B56 regulatory subunit (Hertz et al., 2016; Wang X. et al., 2016). These interactions are strengthened by phosphorylation within and around this motif, which for the case of the BUBR1-KARD, is mediated by Cdk1 and Plk1 (Elowe et al., 2007; Huang et al., 2008; Suijkerbuijk et al., 2012; Kruse et al., 2013; Wang J. et al., 2016; Wang X. et al., 2016) (arrow 8 in Figure 1). Note that Figure 1 only depicts Plk1 phosphorylation because Cdk1 phosphorylations will be discussed in detail later. Furthermore, only one Plk1 site is illustrated, even though two were initially identified (Ser676 and Thr680) (Elowe et al., 2007; Suijkerbuijk et al., 2012; Kruse et al., 2013); this is because phospho-Thr680 does not appear to enhance B56 affinity *in vitro* (Wang J. et al., 2016). The recruitment of PP2A-B56 to kinetochores is needed to supress Aurora B activity enough to allow kinetochores to form initial end-on attachments to microtubules (Suijkerbuijk et al., 2012; Kruse et al., 2013; Xu et al., 2013; Shrestha et al., 2017) (inhibitory arrow 9 from PP2A-B56 in Figure 1). Presumably, this baseline state of low Aurora B activity can then be reduced even further to fully stabilize attachments if a high tension is achieved by biorientation (inhibitory arrow 7 from KT-MT in Figure 1).

As well as inhibiting the effects of Aurora B on the kinetochore-microtubule interface, PP2A-B56 also prevents Aurora B from phosphorylating and inhibiting a PP1 docking motif in the N-terminus of Knl1 (labeled as SILK and RVSF in Figure 1 to reflect their amino acid sequence) (Nijenhuis et al., 2014) (arrows 10 and 11 from Aurora B and PP2A-B56, respectively, in Figure 1). This docking motif allows a PP1-Knl1 complex to: (1) silence SAC signaling (as discussed further below), (2) restrict kinetochore Aurora B activity, and (3) remove PP2A-B56 from kinetochores (Liu et al., 2010; Meadows et al., 2011; Rosenberg et al., 2011; Espeut et al., 2012; Nijenhuis et al., 2014). The removal of PP2A-B56 occurs because PP1-Knl1 dephosphorylates KNL1 on “MELT” repeats to inhibit BubR1 recruitment (arrow 12), and also the BubR1-KARD motif to inhibit BubR1-PP2A interaction (arrow 13, **Figure 1**) (Nijenhuis et al., 2014). This negative feedback loop between PP2A-B56 and PP1-Knl1 has important implications for the SAC and kinetochore-microtubule attachments, which will be discussed in detail later. The ability of PP1-Knl1 to inhibit kinetochore Aurora B activity may be restricted to aligned kinetochores, since it helps to stabilize bipolar attachment at metaphase, but does not seem to be needed for initial chromosome alignment (Liu et al., 2010; Shrestha et al., 2017). One substrate that could be important in this regard is the Ska complex, which is restricted from accumulating at unattached kinetochores by Aurora B (arrow 3), but is then rapidly recruited to bioriented kinetochores by PP1-Knl1 (arrow 14, Figure 1) (Redli et al., 2016). The Ska complex itself also binds to PP1 (Sivakumar et al., 2016), however, it is currently unclear whether this PP1 complex helps to reinforce Aurora B inhibition upon biorientation.

It should be noted that various other PP1 complexes have also been identified at the kinetochore (Yamashiro et al., 2008; Akiyoshi et al., 2009; Kim et al., 2010; Posch et al., 2010; Meadows et al., 2011; De Wever et al., 2014; Häfner et al., 2014; Tang and Toda, 2015; Zhang et al., 2015; Bokros et al., 2016; Duan et al., 2016), and three of these in particular (PP1-Sds22, PP1-Cenp-E and PP1-ASPP1/2), have been implicated in Aurora B and microtubule attachment regulation. These complexes are currently omitted from Figure 1 for various reasons: PP1-ASPP1/2 can be seen to bind to Ndc80 biochemically, but it does not appear to accumulate at kinetochores (Zhang et al., 2015). Sds22 knockdown positively and negatively regulates different Aurora B substrates at the kinetochore (Posch et al., 2010; Wurzenberger et al., 2012; Duan et al., 2016), but this is thought to be due to regulation in the cytoplasm that has downstream consequences for PP1-Knl1 activity (Eiteneuer et al., 2014). Cenp-E binds to PP1, and the Aurora kinases can dynamically regulate this interaction to promote chromosome alignment (Kim et al., 2010). Although PP1-Cenp-E has been implicated in stabilizing microtubule attachments at the kinetochore, it is not currently included in Figure 1 because its effects on KMN network regulation, and on Aurora B substrates in particular, still remain to be elucidated.

## The spindle assembly checkpoint

The SAC holds cells in mitosis until all kinetochores have formed stable attachments to microtubules. In short, unattached kinetochores provide a platform to generate the mitotic checkpoint complex (MCC) which can diffuse throughout the cell to inhibit the anaphase promoting complex/cyclosome (APC/C); an E3 ubiquitin ligase needed for mitotic exit (Musacchio, 2015; Joglekar, 2016; Corbett, 2017). The amount of MCC produced at kinetochores is sufficient to allow just one unattached kinetochore to arrest a cell in mitosis for many hours (Rieder et al., 1995; Dick and Gerlich, 2013). This effectively prevents mitotic exit until each and every kinetochore has formed stable microtubule attachments. The full details of SAC signaling are beyond the scope of this review, however, these are explained in depth in a number of excellent recent articles (London and Biggins, 2014b; Musacchio, 2015; Joglekar, 2016; Corbett, 2017). I will focus here only on the key points needed to describe all of the connections at the KMN network depicted in Figure 1.

The principal kinase that regulates the SAC is Mps1, which localizes to kinetochores by binding to the calponin homology (CH) domains of Ndc80 and Nuf2 (Hiruma et al., 2015; Ji et al., 2015). From here, Mps1 is ideally positioned to phosphorylate key substrates on the KMN network that are needed for MCC formation. The best characterized of these are the “MELT repeats” on Knl1 (London et al., 2012; Shepperd et al., 2012; Yamagishi et al., 2012) (arrow 15, Figure 1), which recruit a pseudo-symmetric Bub1/Bub3-Bub3/BubR1 complex to kinetochores by virtue of an interaction between Bub1/Bub3 and the phosphorylated MELT motif (MELT refers to the consensus amino acid sequence Met-Glu-Leu-Thr) (Yamagishi et al., 2012; Primorac et al., 2013; Vleugel et al., 2013; Zhang et al., 2014; Overlack et al., 2015). Many of these MELT repeats also contain an additional adjacent motif (SHT; for Ser-His-Thr) that becomes a substrate for Mps1 after priming phosphorylation of the MELT (Vleugel et al., 2015). The phosphorylated SHT (SHpT) motif can then collaborate with the phospho-MELT (MELpT) to increase Bub1/Bub3 affinity (this dual phospho-MELT/SHT motif is annotated as “MELT” in Figure 1). Collectively, these Knl1 phosphorylation sites provide a platform for SAC signaling at the kinetochore, because the Bub1/Bub3 complex co-recruits, directly or indirectly, all of the other proteins needed for SAC signaling (BubR1, Mad1, Mad2, Cdc20) (Corbett, 2017). Therefore, Knl1 acts as a scaffold for MCC assembly at the kinetochore (represented by a thick arrow 16 from the Bub complex in Figure 1). Mps1 also directly phosphorylates both Bub1, to regulate Mad1 recruitment, and Mad1 itself to stimulate catalytic assembly of the MCC complex (London and Biggins, 2014a; Moyle et al., 2014; Mora-Santos et al., 2016; Faesen et al., 2017; Ji et al., 2017, 2018; Qian et al., 2017; Zhang G. et al., 2017). Therefore, these additional phosphorylations are represented by arrows 17 and 18 from Mps1 to MCC formation in Figure 1.

In addition to recruiting MCC components, the Bub complex also recruits the kinase Plk1 to the KMN network (Qi et al., 2006; Elowe et al., 2007; Wong and Fang, 2007) where it is able to cooperate with Mps1 to enhance SAC signaling (Von Schubert et al., 2015; Ikeda and Tanaka, 2017). Critical substrates in this regard are the Knl1-MELT repeats, which are phosphorylated by both Mps1 and Plk1 to activate the SAC (arrows 15 and 19, Figure 1) (Espeut et al., 2015; Von Schubert et al., 2015; Ikeda and Tanaka, 2017). Plk1 and Mps1 both have a strong preference for acidic residues at position −2 relative to the phosphoacceptor site (Nakajima et al., 2003; Dou et al., 2011; Santamaria et al., 2011; Oppermann et al., 2012; Hennrich et al., 2013), and therefore they could potentially share many different SAC substrates. Plk1 also phosphorylates Mps1 itself on multiple different sites, which have been proposed to control Mps1 activity Von Schubert et al., 2015; Ikeda and Tanaka, 2017). However, it is unclear whether these phosphorylations directly activate Mps1, therefore this potential link is currently omitted from Figure 1. In addition to stimulating MCC assembly, Plk1 has also been shown to phosphorylate the APC/C co-activator Cdc20, which inhibits the ability of Cdc20 to bind and activate the APC/C (Jia et al., 2016). This phosphorylation is scaffolded by Bub1, which also contributes to Cdc20 inhibition by phosphorylating an additional inhibitory site in the N-terminus of Cdc20 (Tang et al., 2004) (these two phosphorylations are represented by inhibitory arrows 20 and 21 from Bub1 and Plk1 to APC/C^Cdc20^ in Figure 1).

The principal phosphatase that counteracts Mps1 signaling at the kinetochore is PP1. This was first identified as the primary SAC silencing phosphatase in *C. elegans, S. pombe*, and *S. cerevisae* (Pinsky et al., 2009; Vanoosthuyse and Hardwick, 2009; Meadows et al., 2011; Rosenberg et al., 2011; Espeut et al., 2012; London et al., 2012) and later validated as a critical phosphatase in mammalian cells (Nijenhuis et al., 2014). PP1 binds to a variety of different regulatory subunits at the kinetochore (Akiyoshi et al., 2009; Kim et al., 2010; Liu et al., 2010; Meadows et al., 2011; De Wever et al., 2014; Häfner et al., 2014; Tang and Toda, 2015; Sivakumar et al., 2016), however, specifically interfering with PP1-Knl1 interaction is sufficient to give a strong defect in SAC silencing (Meadows et al., 2011; Rosenberg et al., 2011; Espeut et al., 2012; Nijenhuis et al., 2014). Moreover, this is not related to indirect effects on kinetochore-microtubule attachments, because SAC silencing is effectively prevented following Mps1 inhibition in nocodazole (Nijenhuis et al., 2014). Therefore, although additional PP1 complexes maybe important for SAC silencing following kinetochore-microtubule attachment, these cannot substitute for PP1-Knl1 in the absence of microtubules.

A critical feature of PP1-Knl1 is likely to be its precise location at the outer kinetochore, since it lies adjacent to the site of MCC production. Key substrates in this regard are the MELT motifs on Knl1, which are dephosphorylated by PP1-Knl1 to reduce kinetochore Bub1 levels (Nijenhuis et al., 2013; Zhang et al., 2014; Kim et al., 2017) (arrow 12, Figure 1). In Drosophila, the MELT motifs in the Knl1 homolog (Spc105) appear degenerate and dispensable for the SAC (Schittenhelm et al., 2009), but in this case, the activation loop of Mps1 itself is a key substrate of PP1 (Moura et al., 2017). It is unclear, however, whether this is regulated by PP1-Spc105 specifically, and in human cells at least, interfering with PP1-Knl1 directly does not enhance Mps1 activation loop phosphorylation (Nijenhuis et al., 2013). As mentioned previously, there are other key Mps1 substrates in the SAC and it will be important to test whether these are also regulated by PP1-Knl1 or other PP1 complexes at the kinetochore.

At least one other PP1 complex, PP1-Ska1, localizes to the KMN network and regulates Knl1-MELT phosphorylation and SAC silencing (Sivakumar et al., 2016) (arrow 22, Figure 1). This is not essential in the absence of microtubules, since Ska3 depletion removes the Ska complex from kinetochores, but only causes a very mild (10 min) delay in mitotic exit following Mps1 inhibition in nocodazole (Sivakumar et al., 2014; Zhang Q. et al., 2017). However, since the Ska complex is recruited to kinetochores to track growing and shrinking microtubules, one might predict that the Ska1-PP1 axis becomes fully engaged to aid SAC silencing following end-on microtubule attachment. Unfortunately, experiments designed to address this are complicated by the fact that the exact PP1 binding motif in Ska1 is currently unknown, which has necessitated deletion of the entire C-terminal region to prevent PP1 binding (Sivakumar et al., 2016). This region is also critical for microtubule binding (Schmidt et al., 2012; Abad et al., 2014), and therefore until these two key events can be functionally separated, care should be taken when interpreting whether the Ska complex silences the SAC directly (via PP1) or indirectly (via microtubule binding). Furthermore, based on current experiments, it is not possible to dissociate whether the effects of PP1-Ska on the SAC are due to inhibition of Mps1 activity/localisation or downstream dephosphorylation of Mps1 substrates (as discussed in detail later). In summary, Ska-PP1 negatively regulates SAC activity, but unlike PP1-Knl1, it does not appear to be essential for mitotic exit, at least in the absence of microtubules. Perhaps PP1-Knl1 is the primary SAC silencing phosphatase because it can dephosphorylate the MELTs directly, and it can also antagonize Aurora B to recruit Ska1-PP1 to kinetochores, thereby indirectly antagonizing the SAC when kinetochores couple to dynamic microtubules tips (arrows 12 and 14, Figure 1).

It is important to point out that additional kinetochore PP1 complexes have also been implicated in SAC silencing in other species. In budding yeast FIN targets PP1 to kinetochores, but this is principally involved in keeping the SAC silenced at anaphase when Cdk1 activity is lost (Akiyoshi et al., 2009; Bokros et al., 2016). In fission yeast, the kinesin-8 motors, Klp5 and Klp6, bind to the Ndc80 loop region and help PP1-Knl1 to silence the SAC (Meadows et al., 2011; Tang and Toda, 2015). However, the kinesin-8 homolog in human cells, KIF18A, does not appear to regulate SAC silencing, even though it can bind to PP1 and target it to kinetochores (De Wever et al., 2014; Häfner et al., 2014). At this stage, therefore, Kinesin-8 motors are omitted from Figure 1, since their effects on the KMN network may be specific to fission yeast. This is an important issue to resolve in future.

At least one other KMN-localized phosphatase that is critical for SAC silencing in human cells is BubR1-bound PP2A-B56, because BubR1 mutations that prevent PP2A-B56 binding delay SAC silencing and Knl1-MELT dephosphorylation (Espert et al., 2014; Nijenhuis et al., 2014). PP2A-B56 can directly dephosphorylate the MELT motifs *in vitro*, however, whether it does this directly or indirectly via PP1-Knl1 (arrow 11, Figure 1) *in vivo* is currently a matter of debate (Espert et al., 2014; Nijenhuis et al., 2014). At this stage, I have omitted the direct link from PP2A-B56 to the MELTs from Figure 1 because of one important finding: specific inhibition of PP1-Knl1 prevents MELT dephosphorylation and SAC silencing following MPS1 inhibition in nocodazole, but actually enhances PP2A-B56 at the kinetochore (due to increased BubR1 levels and KARD phosphorylation; arrows 12 and 13 in Figure 1) (Nijenhuis et al., 2014). It is hard to explain why the MELTs are not dephosphorylated under these conditions if PP2A-B56 can work directly. BUBR1-bound PP2A-B56 has, however, been directly linked to at least two other sites needed for SAC signaling at the kinetochore. It can antagonize Plk1-dependent phosphorylation of Cdc20 (Craney et al., 2016; Jia et al., 2016) and Mps1 dependent phosphorylation of Bub1 (Qian et al., 2017) (arrows 23 and 24, Figure 1). It is important to stress, however, that the influence of the PP1-Knl1 complex on these sites has not been specifically tested, and furthermore, there is also debate about whether PP2A-B56 antagonizes Cdc20 phosphorylation by binding to BubR1 (Craney et al., 2016; Jia et al., 2016) or directly to the APC/C (Lee et al., 2017). Therefore, it will be important in future to carefully evaluate the relative contribution of different phosphatase subcomplexes on the various phosphorylation sites that are critical for SAC signaling, including the key catalytic sites in the C-terminus of Mad1 (Faesen et al., 2017; Ji et al., 2017, 2018).

Another important issue to resolve in future, is whether kinetochore phosphatase activity can be directly regulated at the KMN network. In fission yeast, PP1 and PP2A-B56 are globally inhibited during mitosis, but reactivated sequentially upon mitotic exit (Grallert et al., 2015). These phosphatase complexes clearly need activity to silence the SAC prior to mitotic exit, therefore, it would be interesting to test whether localized reactivation may occur earlier at the kinetochore. Clustering of PP1 and PP2A-B56 molecules on Knl1 could potentially stimulate this reactivation by enhancing *trans*-dephosphorylation of inhibitory phosphorylation sites (Grallert et al., 2015). At least one protein that is thought to regulate PP2A-B56 activity at kinetochores is Bod1. Bod1 localizes to Ndc80 and is able to inhibit BubR1-bound PP2A-B56 (Porter et al., 2013; Schleicher et al., 2017) (arrow 25, Figure 1). PP1-Knl1 activity may also be modulated by various accessory proteins (Posch et al., 2010; Eiteneuer et al., 2014; Duan et al., 2016), however, this regulation is omitted from Figure 1, because it is thought to occur in the cytoplasm and not at kinetochores. It will be important in future to determine whether these, or other inhibitory pathways, are shut down following chromosome biorientation to enhance kinetochore phosphatase activity and promote SAC silencing.

A final important issue regarding SAC silencing concerns its regulation by kinetochore-microtubule attachment status. As mentioned previously, once stable kinetochore-microtubule attachments have formed, localized SAC signaling must be rapidly extinguished. There are at least three mechanisms that contribute to this rapid silencing. Firstly, microtubule attachment inhibits Mps1 activity, either by displacing the kinase from kinetochores, as demonstrated in human cells (Hiruma et al., 2015; Ji et al., 2015), or by spatially separating Mps1 from its key SAC substrates, as demonstrated in budding yeast (Aravamudhan et al., 2015) (inhibitory arrow 26 to Mps1 in Figure 1). Secondly, if these attachments generate tension, then Aurora B is inhibited at the kinetochore, which enhances PP1-Knl1 activity to antagonize the SAC signal (arrows 7 and 10, Figure 1). This could explain why Aurora B activity is needed to prevent the premature removal of SAC proteins from kinetochores (Gurden et al., 2016). Finally, the attached microtubules provide a highway onto which dynein motors can travel to transport key SAC proteins away from kinetochores toward the spindle poles (not depicted on Figure 1) (Howell et al., 2001; Wojcik et al., 2001; Mische et al., 2008; Sivaram et al., 2009). Therefore, the effects of microtubules combine to shut down the upstream kinase input, switch on the antagonizing phosphatases, and strip the remaining SAC signal away. This essentially leads to a responsive SAC signal that can shut off at kinetochores within seconds following microtubule attachment.

## The role of CDK1 in regulating kinetochore-microtubule attachments and the SAC

**Figure 2:**
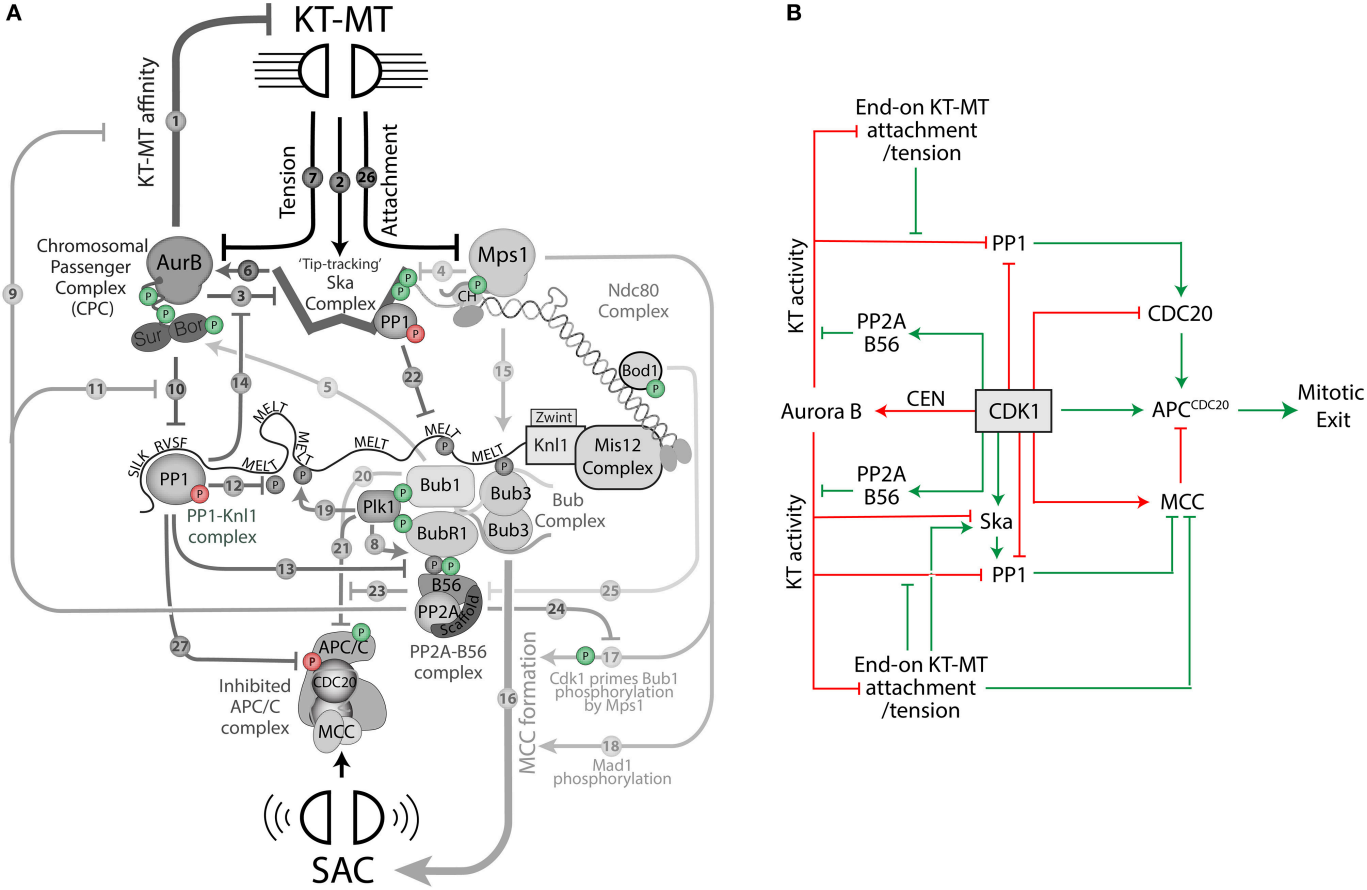
A schematic to depict how Cdk1 regulates the KMN network to control mitotic exit. **(A)** Colored phosphate symbols indicate either positive (green) or negative (red) regulation from Cdk1. **(B)** An arrow-style schematic to depict how Cdk1 promotes (green arrows) and inhibits (red arrows) mitotic exit.

The scheme in Figure 1 incorporates all the main kinases and phosphatases that act at the KMN network with the exception of just one: Cdk1. The reason for this omission is that Cdk1 has been linked to every single node in the network, which would lead to a very complicated picture if incorporate into the same arrow-style schematic. Instead, having discussed the core network, I will now overlay Cdk1 regulation on top of this by the addition of phosphate symbols to the various components (see Figure 2A; a green phosphate symbol indicates a direct activating input from Cdk1, whereas a red phosphate indicates a direct inhibitory input). This figure should be referenced in conjunction with the text below, where each of these regulatory inputs will be briefly explained. The text will also contain additional indirect regulation that is relevant for KMN regulation, but it not depicted in Figure 2 because it does not occur locally at the KMN network.

Cdk1 phosphorylates the APC/C to enhance its affinity for Cdc20, thereby increasing APC/C^Cdc20^ activity (Shteinberg et al., 1999; Kramer et al., 2000; Rudner and Murray, 2000; Kraft et al., 2003; Fujimitsu et al., 2016; Qiao et al., 2016; Zhang et al., 2016; Figure 2A). To prevent immediate mitotic exit, however, Cdk1 then initiates a series of events that collaborate to antagonize APC/C^Cdc20^ by two general mechanisms:

1. *Stimulation of MCC production* (see Figure 2A). Cdk1 elevates Mps1 activity by direct phosphorylation on Ser283. This residue is located outside of the catalytic domain, but when phosphorylated, is able to amplify Mps1 activity without affecting its kinetochore recruitment (Morin et al., 2012). Cdk1 also helps Mps1 to initiate SAC signaling in multiple ways: It phosphorylates Bub1 (on Ser459) to prime further phosphorylation by Mps1 on Thr461 (arrow 17), which together help to promote Mad1 interaction and MCC formation (Chen, 2004; Ji et al., 2017; Qian et al., 2017; Zhang Q. et al., 2017). In addition, Cdk1 phosphorylates Bub1 (on Thr609) and BubR1 (on Thr620) to create docking sites for the polo-box domains (PBDs) of Plk1 (Qi et al., 2006; Elowe et al., 2007; Wong and Fang, 2007). As discussed earlier, this helps to enhance SAC signaling by allowing Plk1 to co-operate with Mps1 to phosphorylate the Knl1-MELT motifs (Espeut et al., 2015; Von Schubert et al., 2015; Ikeda and Tanaka, 2017) (arrow 19). Finally, there are also at least two other ways that Cdk1 can influence Plk1 activity at kinetochores: (1) Cdk1 primes USP16 for phosphorylation and activation by Plk1, which establishes a positive feedback loop that helps to maintain Plk1 on kinetochores by antagonizing its CUL3–KLHL22-mediated ubiquitylation and removal (Beck et al., 2013; Zhuo et al., 2015), (2) Cdk1 phosphorylates the MYPT1-PP1 complex to enhance Plk1 interaction and decrease Plk1 activity (Yamashiro et al., 2008; Dumitru et al., 2017). Although these connections can both impact on kinetochore-localized Plk1 activity, they are not currently annotated on Figure 2A because it is still unclear whether this regulation occurs locally at the KMN network. These are important issues to resolve in future.
2. *Direct Inhibition of Cdc20* (see Figure 2A). Cdk1 phosphorylates Cdc20 to negatively regulate its ability to bind and activate the APC/C (Yudkovsky et al., 2000; Labit et al., 2012; Hein and Nilsson, 2016). In addition, Cdk1 activity directly and indirectly recruits Plk1 and Bub1 to kinetochores, as discussed above, and these kinases cooperate to phosphorylate additional sites in the N-terminus of Cdc20 to inhibit its activity (Tang et al., 2004; Jia et al., 2016) (arrows 20 and 21).

As well as stimulating both of these inhibitory pathways, Cdk1 also antagonizes the phosphatase complex that is eventually needed to reverse these pathways and activate the APC/C: PP1-KNL1. As discussed earlier, PP1-Knl1 dephosphorylates the Knl1-MELT motifs, and perhaps other key SAC substrates, to extinguish MCC production. In addition, it has also been shown to reverse inhibitory Cdk1 phosphorylation on Cdc20 to allow activation of the APC/C upon mitotic exit in *C. elegans* (Kim et al., 2017) (arrow 27 from PP1 to APC/C^Cdc20^ in Figure 2A). Together, this explains why PP1-KNL1 must be inhibited at unattached kinetochores to prevent premature APC/C activation. This inhibition is promoted by Cdk1, both directly and indirectly, because Cdk1 can directly phosphorylate and inhibit the catalytic subunit of PP1 (Dohadwala et al., 1994; Yamano et al., 1994; Kwon et al., 1997; Wu et al., 2009; Grallert et al., 2015), and it can also activate Aurora B to inhibit PP1-KNL1 assembly at kinetochores.

Cdk1 can activate Aurora B in a variety of ways, but most of these converge to help the clustering of CPC molecules at centromeres, which in turn, helps Aurora B to auto-activate in *trans* (Bishop and Schumacher, 2002; Honda et al., 2003; Yasui et al., 2004; Sessa et al., 2005; Kelly et al., 2007; Wang et al., 2011; Zaytsev et al., 2016). Cdk1 directly phosphorylates borealin (or survivin in fission yeast) to promote binding to Shugoshin 1 (Sgo1). This recruits the CPC to Bub1-phosphorylated Histone H2A tails and cohesin rings at the centromere (Kawashima et al., 2010; Tsukahara et al., 2010). Cdk1 additionally phosphorylates T346 on Sgo1 to enhance its binding to cohesin rings (Liu et al., 2013), and in human somatic cells, it helps to enrich this cohesin at the centromere by promoting its loss from chromosome arms (Dreier et al., 2011; Liu et al., 2013; Nishiyama et al., 2013). Another pathway that is important for centromeric CPC recruitment is the Haspin-mediated phosphorylation of Histone H3 tails (H3-pT3), which stimulates their interaction with survivin (Kelly et al., 2007; Wang et al., 2010; Yamagishi et al., 2010; Jeyaprakash et al., 2011; Niedzialkowska et al., 2012). Cdk1 phosphorylates Haspin to prime the binding of its activator Plk1, thereby enhancing H3-T3 phosphorylation (Ghenoiu et al., 2013; Zhou et al., 2014). At the same time, Cdk1 inhibits the H3-T3 phosphatase, PP1-RepoMan, to prevent H3-T3 dephosphorylation (Vagnarelli et al., 2011; Qian et al., 2015). Collectively, these direct and indirect effects of Cdk1 serve to localize and activate the CPC at centromeres.

In addition to regulating centromere recruitment, Cdk1 also phosphorylates INCENP on at least two residues that are functionally important. It phosphorylates Thr59, which prevents premature localisation of the CPC to the spindle midzone and must be dephosphorylated to properly switch off the SAC at anaphase (Goto et al., 2006; Hümmer and Mayer, 2009; Vázquez-Novelle and Petronczki, 2010). Cdk1 also phosphorylates Thr388 in INCENP, which creates a docking site for Plk1 (Goto et al., 2006). This docking site is likely to contribute to cross-talk between Aurora B and Plk1 (Combes et al., 2017), although it is currently unclear whether this affects their respective activities at the KMN network. Interestingly, Plk1 and Aurora B have been proposed to cooperate in a positive feedback loop that enhances their respective activities at the kinetochore (O'connor et al., 2015), therefore, perhaps INCENP phosphorylation could be relevant in this context. Finally, Cdk1 may also help to protect deactivation of Aurora B since it phosphorylates and activates the acetyltransferase TIP60, which can acetylate Aurora B and protect its activation loop from dephosphorylation by PP2A (Mo et al., 2016). TIP60 phosphorylation occurs at the kinetochore and is needed for proper chromosome segregation, but this regulation is currently omitted from Figure 2 because it is unclear whether it helps to maintain Aurora B activity at the KMN network. The balance between PP2A and Aurora B is clearly critical for microtubule attachment and SAC regulation, therefore this is an important issue to resolve in future.

In summary, Cdk1 activates the APC/C but then initiates a whole series of events that converge to inhibit the APC/C and prevent mitotic exit (see Figure 2B). In one important final twist, Cdk1 attempts to override this APC/C inhibition and induce mitotic exit by stimulating at least two other pathways at the kinetochore: Cdk1 phosphorylates BubR1 (on Ser670) and Ska3 (on Thr358 and Thr360) to recruit PP2A-B56 and the Ska complex to kinetochores (Huang et al., 2008; Suijkerbuijk et al., 2012; Kruse et al., 2013; Zhang Q. et al., 2017). Importantly, the ability of these two pathways to promote mitotic exit is enhanced by the formation of end-on microtubule attachments (green arrows in Figure 2B). Therefore, Cdk1 jointly stimulates both mitotic arrest and mitotic exit, but it hands the control over to microtubules, which can determine whether kinase or phosphatase activities predominate at the kinetochore (as discussed in detail later). Part of this balance may also be controlled by Bod1, which binds to NDC80 and is phosphorylated by Cdk1 to inhibit PP2A-B56 (Porter et al., 2013; Schleicher et al., 2017; Figure 2A). It will be interesting to determine whether microtubule attachment or tension can modulate Bod1 localisation or phosphorylation.

## The implications of cross-talk at the KMN network

Inhibition of any one of the enzymes depicted in Figure 2A will produce knock-on effects for all the others. This has inevitable consequences for both kinetochore-microtubule attachments and the SAC, and therefore, instead of viewing these enzymes in isolation, it is probably safer, and more accurate, to view them as part of a single network that can co-regulate two key mitotic processes. In this respect, it is critical to understand the implications of all this cross-talk. One only needs to trace some of the arrows in Figure 2A, to see that this network is rich in various forms of feedback and feedforward regulation, and yet very little is currently known about what this could mean for microtubule attachments and the SAC.

I would now like to focus on each enzyme individually to discuss some of these issues. In particular, I will discuss how coactivation of kinases and phosphatases may help to produce dynamic signals that can quickly respond to changes in microtubule occupancy. I will also highlight how the multiple connections within this network inevitably complicates interpretations about the direct effects of each particular enzyme. This may have contributed to some controversies within the field, and therefore after highlighting these issues, I will finish by discussing some general experimental approaches that may help to resolve some of the questions that remain. Before I focus in on each of the enzymes, however, it is first important to stress that microtubules themselves can also be a general source of unwanted cross-talk.

## Indirect effects of microtubule attachments on the SAC

As discussed previously, kinetochore-microtubule attachments shut down the SAC signal in many different ways (Etemad and Kops, 2016). Therefore, experiments designed to probe the SAC directly should be interpreted extremely cautiously if microtubules are present that could indirectly impact on SAC strength. In fact, as pointed out previously by others (Khodjakov and Rieder, 2009), in many instances it is best to avoid this situation by using microtubule depolymerizing agents, such as nocodazole, to ensure that kinetochores remain in an unattached state throughout the assay. One common reason for preserving microtubules is to achieve a state of submaximal SAC strength. For example, the use of taxol to stabilize microtubules or Eg5 inhibitors to induce monopolar spindles, produces fewer unattached kinetochores to signal to the SAC in comparison to nocodazole (Collin et al., 2013). The result is that SAC strength is reduced, and defects within the SAC signaling network can produce more penetrant phenotypes. However, if these defects also affect kinetochore-microtubule stability then this will inevitably produce indirect effects on the SAC. Furthermore, as Figure 1 demonstrates, all of the validated SAC regulators can impinge on kinetochore-microtubule attachments either directly or indirectly. Therefore, to avoid this cross-talk from confounding interpretations about the SAC, a useful alternative approach is to combine nocodazole with a low dose of an Mps1 inhibitor to sensitize the SAC whilst still keeping all kinetochores free from microtubule attachments. This type of experiment was crucial for characterizing the role of Aurora B in the SAC.

### Aurora B

Aurora B had long been suspected to activate the SAC directly, primarily because Aurora B inhibition weakened the SAC in the nocodazole (Kallio et al., 2002; Ditchfield et al., 2003; Hauf et al., 2003; Petersen and Hagan, 2003; Vader et al., 2007; Vanoosthuyse and Hardwick, 2009). This was controversial, however, since others had suggested that residual microtubules at the chosen nocodazole concentrations may still silence the SAC indirectly (Yang et al., 2009). Furthermore, even if Aurora B did signal directly to the SAC, then it was unclear at what level Aurora B exerted its control. These issues were resolved using sensitized assays employing a high dose of nocodazole combined with partial Mps1 inhibition or Ndc80 depletion. These experiments demonstrated that Aurora B acts at the apex of SAC signaling to establish Mps1 activity at kinetochores (Santaguida et al., 2011; Saurin et al., 2011).

**Figure 3:**
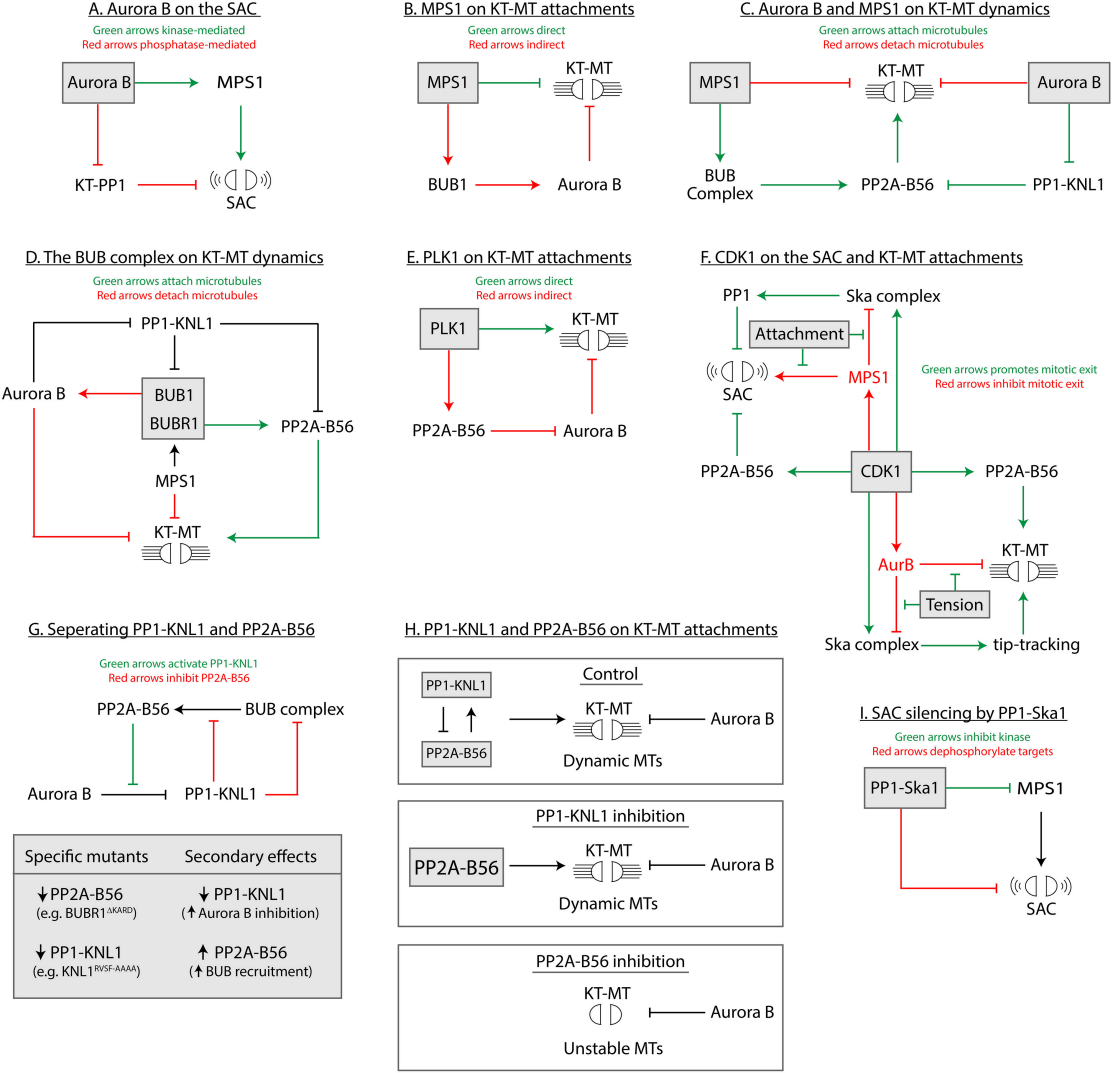
Models focussed on each enzyme at the KMN network **(A–I)** to depict the different types of direct and indirect regulation.

This is presumed to be via the direct recruitment of Mps1 to kinetochores because Aurora B inhibition in nocodazole reduces kinetochore Mps1 and weakens the SAC, and artificial tethering of Mps1 to Mis12 can rescue these SAC defects (Jelluma et al., 2010; Santaguida et al., 2011; Saurin et al., 2011; Nijenhuis et al., 2013). However, an arrow between Aurora B and Mps1 is omitted from Figure 1 because there is currently no direct mechanistic evidence to support this link. In fact, herein lies an important note of caution: if Aurora B inhibits phosphatase complexes that antagonize Mps1 (PP1-Knl1 and PP1-Ska; arrows 10 and 3 in Figure 1), then this “double negative” input could well explain the “positive” effect of Aurora B on the SAC (Figure 3A). Or to put this another way, Aurora B inhibition could simply enhance kinetochore phosphatase activity to antagonize Mps1 and silence the SAC. The fact that tethering Mps1 to kinetochores overrides the effect of Aurora B inhibition does not preclude this hypothesis, because high levels of kinetochore-Mps1 could simply counterbalance high phosphatase activity to preserve the SAC. In fact, If Aurora B acts exclusively via PP1, then it may be easier to rationalize why Aurora B inhibition in nocodazole is so well tolerated. Otherwise, if Aurora B inhibition increases SAC silencing and decreases Mps1 activity, it is difficult to explain why it does not also rapidly extinguish the SAC in the absence of microtubules. To resolve these issues in future, it will be important to clarify exactly how kinetochore Mps1 recruitment is regulated and to determine whether the input from Aurora B is direct or indirect (via PP1, for example; Figure 3A). It will also be important to develop direct reporters of Mps1 activity because simply using Mps1 kinetochore levels as a surrogate for its activity could be misleading, especially since Mps1 activity negatively regulates its own kinetochore accumulation (Hewitt et al., 2010; Jelluma et al., 2010; Santaguida et al., 2010; Von Schubert et al., 2015).

### Mps1

In addition to activating the SAC, Mps1 also regulates kinetochore-microtubule attachments because knockdown or inhibition of Mps1 prevents the correction of maloriented sister chromatids. Exactly how this occurs, however, has been a matter of considerable debate, with evidence that Mps1 works both dependently and independently of Aurora B (Maure et al., 2007; Jelluma et al., 2008; Kwiatkowski et al., 2010; Maciejowski et al., 2010; Santaguida et al., 2010; Sliedrecht et al., 2010). At least some of the Aurora B-dependent action can be explained by the kinetochore recruitment of Bub1, which enhances the centromeric localisation of Aurora B via Sgo1 (Hindriksen et al., 2017; Figure 3B). In agreement with this, tethering of Aurora B to centromeres is sufficient to partially rescue chromosome alignment following Mps1 inhibition (Van Der Waal et al., 2012). In contrast, the Aurora B-independent effects may be explained by direct phosphorylation of the Ska complex, which regulates its ability to track dynamic microtubule ends (Maciejowski et al., 2017).

Mps1 and Aurora B activities are counteracted at the outer kinetochore by PP2A-B56 (Foley et al., 2011; Maciejowski et al., 2017). Importantly, both of these kinases also help to elevate kinetochore PP2A-B56 levels: Mps1 enhances PP2A-B56 recruitment (via the Bub complex) and Aurora B inhibits its removal (via PP1-Knl1; Figure 1). Therefore, Aurora B and Mps1 phosphorylate substrates to remove kinetochore-microtubule attachments, and at the same time, they recruit a phosphatase that attempts to stabilize these attachments (Figure 3C). This type of “paradoxical regulation,” also called incoherent feedforward regulation (Hart and Alon, 2013), induces cycles of phosphorylation and dephosphorylation on individual molecules, which in turn, can enhance substrate responsiveness (i.e., the speed by which these substrates can change phosphorylation state; see Gelens and Saurin, 2018). Therefore, the continual phosphorylation and dephosphorylation of kinetochore targets may help to generate a dynamic kinetochore-microtubule interface that can rapidly respond to changes in microtubule occupancy. The phospho-RVSF and phospho-MELT are two examples of dynamic kinetochore substrates needed for SAC responsiveness (Nijenhuis et al., 2014), and it will be interesting to test whether phosphorylation sites that regulate microtubule attachment, such as those on Ndc80, are similarly dynamic.

If balanced phosphorylation and dephosphorylation helps to keep the microtubule interface dynamic, then it will be important to understand how this balance changes over time following the establishment of kinetochore-microtubule attachments and tension. Here, subtle differences in timing could have important physiological consequences. For example, although Mps1 activity falls rapidly following microtubule attachment (Aravamudhan et al., 2015; Hiruma et al., 2015; Ji et al., 2015), PP2A-B56 will persist at kinetochores until its recruitment sites are dephosphorylated, which may provide the time window needed for PP2A-B56 to reverse Mps1 signals. If one considers how the same kinase-phosphatase balance changes over time in other contexts, it may help to solve some current controversies. For example, if Mps1 is re-recruited to attached kinetochores, via Mis12, then microtubule attachments become rapidly destabilized (Maciejowski et al., 2017). However, if Mps1 is maintained at the same location from the start of mitosis then these attachments can apparently form normally (Jelluma et al., 2010). This may, as previously suggested (Maciejowski et al., 2017), relate to differences in kinetochore Mps1 levels, or alternatively, it may relate to differences in PP2A-B56 localization. For example, following constitutive Mis12-Mps1 expression, PP2A-B56 is expected to be maintained at kinetochores during metaphase to counterbalance Mps1 activity, perhaps explaining why these attachments can still become stabilized. However, a time-lag in PP2A-B56 localization following Mps1 re-recruitment may cause these bipolar attachments to become rapidly destabilized before PP2A is present to counterbalance Mps1 activity.

A similar delay in PP2A recruitment occurs upon mitotic entry because BubR1 is excluded from the nucleus in prophase, which in this case, allows MPS1 to rapidly establish the SAC before a phosphatase is recruited to dampen MPS1 signaling in prometaphase (Nijenhuis et al., 2014). This biphasic response has been proposed to function as an attachment-independent biochemical timer that establishes the SAC in prometaphase by temporarily enhancing Bub1-Mad1 interaction, before RZZ-Mad1 interaction is able to take over and sustain the SAC in a microtubule-dependent manner (Qian et al., 2017). It is important to point out, however, that PP2A only supresses KNL1-MELT phosphorylation during a prolonged prometaphase arrest (to approximately 30% of maximal levels; see Nijenhuis et al., 2014). This is likely to reflect rapid phosphorylation-dephosphorylation of individual molecules, which may result in supressed MCC production, or, as hypothesized recently (Gelens and Saurin, 2018), could even help to enhance SAC signaling by promoting the rapid binding and release of MCC components to and from the kinetochore.

### Bub1

Bub1 is a critical scaffold for the SAC, because it recruits BubR1, Mad1/Mad2 and Cdc20 to Knl1 to help generate the MCC (Figure 1). Although its direct catalytic activity appears largely dispensable for SAC signaling (Baron et al., 2016), it does still co-recruit Plk1 which can inhibit Cdc20 (Jia et al., 2016) and collaborate with Mps1 to phosphorylate the Knl1-MELT motifs (Espeut et al., 2015; Von Schubert et al., 2015; Ikeda and Tanaka, 2017). The Bub complex is also a key integrator of kinetochore-microtubule attachment signals: Bub1 elevates Aurora B activity by recruiting the CPC to centromeres (Hindriksen et al., 2017), but at the same time, BubR1 scaffolds PP2A-B56 at kinetochores to antagonize Aurora B activity (Suijkerbuijk et al., 2012; Kruse et al., 2013; Xu et al., 2013; Figures 3D). As discussed above, this type of paradoxical regulation may help to maintain dynamic phosphorylation of the kinetochore-microtubule interface. In this context, the Bub complex is a potentially vulnerable node in the KMN network because altering this kinase-phosphatase balance could alter phosphorylation dynamics and give rise to the type of division errors that are typical seen in tumors with chromosomal instability. This may explain why modulating the Bub1/BubR1 ratio at kinetochores affects kinetochore-microtubule dynamics and tumorigenesis (Lampson and Kapoor, 2005; Jeganathan et al., 2007; Bohers et al., 2008; Baker et al., 2009, 2013; Suijkerbuijk et al., 2010; Ricke and Van Deursen, 2011; Ricke et al., 2011). This is a hypothesis that is discussed in detail elsewhere (Cordeiro et al., 2018).

### Plk1

As discussed above, Plk1 and Mps1 cooperate to phosphorylate the MELT motifs on KNL1 and activate the SAC (Espeut et al., 2015; Von Schubert et al., 2015; Ikeda and Tanaka, 2017). Considering the substrate specificities of Plk1 and Mps1 are largely overlapping (Nakajima et al., 2003; Dou et al., 2011; Santamaria et al., 2011; Oppermann et al., 2012; Hennrich et al., 2013), it will be important to determine whether Plk1 can similarly phosphorylate other key Mps1 sites on Knl1 (Vleugel et al., 2015), Bub1 (London and Biggins, 2014a; Moyle et al., 2014; Mora-Santos et al., 2016; Ji et al., 2017; Qian et al., 2017; Zhang Q. et al., 2017) and Mad1 (Faesen et al., 2017; Ji et al., 2017, 2018). The fact that Plk1 functionally substitutes for Mps1 in *C. elegans*, which lack Mps1 altogether, implies that Plk1 may support more than simply Knl1-MELT phosphorylation (Espeut et al., 2015). Furthermore, it is currently unknown whether Mps1 can similarly substitute for Plk1 at the KMN network, and in particular, whether it can phosphorylate the BubR1-KARD motif and the inhibitory Plk1 site on Cdc20 (Elowe et al., 2007; Jia et al., 2016). Finally, if these kinases are truly cooperative, then it is also important to consider how Plk1 is rapidly inhibited following kinetochore-microtubule attachment. The interaction between Ndc80 and microtubules abruptly switches off kinetochore Mps1 activity to silence the SAC (Aravamudhan et al., 2015; Hiruma et al., 2015; Ji et al., 2015), but it is not clear why Plk1, which is localized all along Knl1 (via interaction with the Bub complex), cannot continue to phosphorylate the MELTs and sustain SAC signaling at this time. Perhaps the Plk1 docking sites on Bub1/BubR1 are other critical targets of PP1-Knl1 and/or PP2A-B56?

As well as supporting the SAC, Plk1 also helps to stabilize initial kinetochore-microtubule attachments during prometaphase (Liu et al., 2012). This may be an indirect effect of Aurora B inhibition, because as stated above, Plk1 enhances the interaction between PP2A-B56 and BubR1. Alternatively, Plk1 has been proposed to work independently of Aurora B in this regard (Liu et al., 2012; Figure 3E). Plk1 can affect kinetochore-microtubule stability in many different ways (Li et al., 2010; Bader et al., 2011; Hood et al., 2012; Maia et al., 2012; Kakeno et al., 2014; Shao et al., 2015), from a variety of different kinetochore subcomplexes (Nishino et al., 2006; Elowe et al., 2007; Amin et al., 2014; Kim et al., 2014; Dumitru et al., 2017; Ehlen et al., 2018). Therefore, these multiple different inputs from Plk1 may help to resolve the puzzling observations that Plk1 inhibition can either destabilize (Sumara et al., 2004; Lénárt et al., 2007) or stabilize (Foley et al., 2011) kinetochore-microtubule attachments under different conditions.

### Cdk1

Cdk1 can positively or negatively regulate all of the enzymes present on the KMN network (Figure 2A). Interestingly, there is a pattern to this regulation when viewed in relation to the APC/C. Cdk1 activates APC^Cdc20^ at the start of mitosis, but then initiates a series of events that cooperate to inhibit APC/C^Cdc20^ and prevent mitotic exit. Specifically, Cdk1 inhibits Cdc20 directly, promotes MCC formation, activates Aurora B to destabilize kinetochore-microtubule attachments, and inhibits the SAC phosphatase PP1, both directly, and indirectly via Aurora B (all red arrows from Cdk1 in Figure 2B). These multiple regulatory inputs from Cdk1 most likely help to ensure that the SAC cannot be reactivated once Cyclin B is degraded and chromosome segregation has been initiated (Vázquez-Novelle et al., 2010, 2014; Rattani et al., 2014). They could also help to regulate the SAC locally at kinetochores *during* mitosis, because Cyclin B/Cdk1 has been observed to localize specifically to unattached kinetochores (Chen et al., 2008). Removal of Cyclin B from kinetochores upon microtubule attachment could potentially reduce local Cdk1 activity to silence the SAC signal and/or inhibit kinetochore Aurora B. It will be interesting to test if any of the Cdk1 phosphorylation sites at the KMN network are sensitive to microtubule attachment status (Figure 2A). It also important to note that Cyclin A/Cdk1 can preferentially phosphorylate a subset of Cdk1 substrates to regulate kinetochore-microtubule turnover (Kabeche and Compton, 2013; Dumitru et al., 2017). Cyclin A is degraded early during prometaphase (Den Elzen and Pines, 2001), which implies that the balance of phosphorylation on Cdk1 substrate at the KMN network may change dynamically as mitosis progresses.

Although a key role of Cdk1 is to arrest the mitotic state, there are at least two Cdk1 substrates at the KMN network that promote mitotic exit instead: Cdk1 phosphorylates BubR1 and Ska3 to recruit PP2A-B56 and the Ska complex to kinetochores (Huang et al., 2008; Suijkerbuijk et al., 2012; Kruse et al., 2013; Xu et al., 2013; Zhang Q. et al., 2017). Both of these pathways function to stabilize microtubule attachments and shut down the SAC (green arrows from Cdk1 in Figure 2B). Therefore, as illustrated in Figure 3F, Cdk1 promotes both mitotic arrest (red arrows), and mitotic exit (green arrows), but allows the output to be decided by the level of kinetochore-microtubule attachment/tension, which can inhibit the kinases and activate PP1 at the kinetochore. It is unclear how Aurora B activity is modulated by tension, but any model of tension-sensing would also need to integrate the regulation of PP2A-B56, PP1-Knl1 and PP1-Ska1. It is not immediately obvious how these phosphatases will behave in time following microtubule attachment or tension, because they are co-regulated by multiple attachment sensitive kinases (Cdk1, Plk1, and Aurora B), and crucially, they also cross-regulate each other.

### Kinetochore phosphatases

As discussed previously, the cross-regulation between PP1-Knl1 and PP2A-B56 at the KMN network complicates the interpretations about exactly which phosphatase regulates the SAC and kinetochore-microtubules attachments directly. The problem stems from the fact that PP2A-B56 enhances PP1-Knl1 accumulation (by inhibiting Aurora B), and therefore mutations that abolish kinetochore PP2A-B56 also reduce PP1-Knl1 (Nijenhuis et al., 2014; Figure 3G). To uncouple the relative contribution of each phosphatase it is important to compare this situation to one in which PP1-Knl1 is abolished directly: because this actually *enhances* kinetochore PP2A-B56 (due to the negative regulation of both Knl1-MELT and BubR1-KARD phosphorylation by PP1-Knl1) (Figure 3G). If both mutants have the same phenotypic effect, as they do for Knl1-MELT phosphorylation, then it is likely that Knl1-PP1 is directly responsible. However, to prove this definitively, it is important to show that the effects of PP2A-B56 inhibition are recovered if PP1-Knl1 is rescued at the kinetochore. It will be important to determine how Knl1-MELT phosphorylation behaves under these conditions.

When characterizing phosphatase inputs at the KMN network it is advisable to perform specific mutations that inhibit local phosphatase recruitment without affecting total phosphatase levels. This minimizes the indirect effects and may also produce more penetrant phenotypes, in comparison to siRNA-mediated depletion, for example. This may explain why Knl1-MELT dephosphorylation was strongly affected by targeting Knl1-PP1 directly, but not by depleting PP1 catalytic subunits (Espert et al., 2014; Nijenhuis et al., 2014). Another important SAC substrate, Bub1-pSer461, was recently shown to be elevated by BubR1 mutations that reduce kinetochore PP2A-B56, but unaffected by depletion of PP1 catalytic subunits (Qian et al., 2017). These data should be interpreted cautiously until this site is assessed following Knl1 mutations that abolish PP1-Knl1 directly. If Bub1-pSer461 is a specific substrate for PP2A-B56, then phosphorylation should be unaffected, or even decreased, under these conditions. Mps1 phosphorylates many other targets on the KMN network to activate the SAC and it will be important in future to carefully dissect which phosphatase complex(es) reverse these phosphorylations.

One other key functional difference between PP1-KNL1 and PP2A-B56 relates to their ability to regulate kinetochore-microtubule attachments. As discussed previously, removing kinetochore PP2A-B56 prevents the formation of initial kinetochore-microtubule attachments due to elevated Aurora B activity (Foley et al., 2011; Suijkerbuijk et al., 2012; Kruse et al., 2013; Xu et al., 2013). This does not appear to be the case when PPI-Knl1 is removed (Liu et al., 2010; Shrestha et al., 2017), which suggests that PP2A-B56 is the primary phosphatase responsible for antagonizing Aurora B prior to the establishment of tension in human cells. However, these data may still be consistent with a role for both PP1-Knl1 and PP2A-B56 in antagonizing Aurora B, since the negative effects of PP1-Knl1 mutation could simply be masked by a compensatory increase in kinetochore PP2A-B56 (as a result of phosphatase cross-talk; see Figure 3H). In agreement with this hypothesis, rescuing PP1-Knl1 following PP2A-B56 depletion is sufficient to reduce Aurora B activity and improve chromosome alignment (Nijenhuis et al., 2014). It will be important to quantify kinetochore Aurora B substrates directly under these different conditions.

A final issue regarding phosphatase specificity involves the PP1-Ska1 complex and how it functions to silence the SAC. Specifically interfering the PP1-Ska, by deleting the Ska1 C-terminal region, elevates Knl1-MELT phosphorylation and Bub1 recruitment in nocodazole (Sivakumar et al., 2016). However, the impact on the activity of Mps1 itself was not determined, and therefore PP1-Ska could either inhibit Mps1 activity, dephosphorylate the Knl1-MELT motifs, or target both Mps1 and Knl1 (Figure 3I). The Ska complex binds directly to the Ndc80 complex, although it is currently unclear whether this is mediated by the Ndc80 tail region, CH domain, or the coiled-coil regions of Ndc80 and Nuf2 (Zhang et al., 2012, 2018; Cheerambathur et al., 2017; Janczyk et al., 2017; Helgeson et al., 2018). In fact, the presence of multiple discrete binding interfaces, has prompted others to speculate that Ska may be present in different subcomplexes bound to Ndc80 (Zhang et al., 2018). Irrespective of whether these subcomplexes exist, it is likely that the PP1-Ska complex is well positioned to regulate Mps1 activity or localization, since Mps1 binds to the CH domains in Ndc80/Nuf2 (Nijenhuis et al., 2013; Hiruma et al., 2015; Ji et al., 2015). The Ska complex accumulates at a time when Mps1 activity needs to be silenced (i.e., following end-on kinetochore microtubule attachment), and PP1 is responsible for dephosphorylating the activation loop of Mps1 in drosophila (Moura et al., 2017). Considering all of these points, it will be interesting to test whether PP1-Ska1 contributes to rapid Mps1 inhibition following microtubule attachment in human cells. If it does, then it may also explain why Aurora B inhibition, which enhances Ska accumulation at kinetochores (Chan et al., 2012; Sivakumar and Gorbsky, 2017), also inhibits Mps1 localisation and activity (Jelluma et al., 2010; Santaguida et al., 2011; Saurin et al., 2011; Nijenhuis et al., 2013).

## Experimental approaches to understand signaling specificity at the outer kinetochore

To understand signaling specificity at the KMN network, it is important to be certain of the direct substrates for each particular enzyme. These can be identified *in vitro* using purified components, however, these results should also be confirmed within the context of the KMN network since specificity *in vivo* is determined by many additional factors, such as relative protein levels and substrate access/availability. For example, although Plk1 and Mps1 may have similar substrate specificities *in vitro*, from their exact locations on the KMN network they may have restricted access to only a subset of these substrates. This access will be defined by the relative geometry of the KMN network and the extent of kinase inactivation following release from its primary docking site(s). Does Mps1 need to be docked on Ndc80 to phosphorylate substrates or can it be released to form a gradient of activity around Ndc80? If it can be released, then what deactivates Mps1 and does Ndc80 binding promote clustering and reactivation (Kang et al., 2007; Mattison et al., 2007; Hewitt et al., 2010)? If it must be docked to be active, for example to relieve autoinhibition (Combes et al., 2018), then do all Ndc80 molecules have similar access to substrates? Ndc80 recruitment to the KMN network is dependent on two separate branches, controlled by Cenp-T and Cenp-C (Musacchio and Desai, 2017), and even in the same Cenp-T branch, some Ndc80 complexes bind within the KMN network, whereas others tether directly to Cenp-T (Nishino et al., 2013; Huis in'T Veld et al., 2016). Perhaps this means that Mps1 activity toward substrates on Knl1 may not be homogenous across the KMN network, or alternatively, and as suggested previously by others (Samejima et al., 2015), perhaps Mps1 only binds to a subset of Ndc80 molecules?

The question of *in vivo* specificity is particularly important with respect to the phosphatases. PP1 and PP2A have broadly overlapping substrate specificities *in vitro* (Ingebritsen and Cohen, 1983; Cohen, 1989), and yet they localize to a very similar molecular space on the KMN network, but still manage to control distinct substrates and processes (Nijenhuis et al., 2014). It is unclear whether this is due to subtle catalytic preferences, relative geometries, or both, but switching the position of PP1 and PP2A at the kinetochore may help to address this issue. It should also be noted that specificity can also occur between phosphatase complexes within the same subclass. PP2A-B56, for example, exists as at least five different isoforms encoded by separate genes (Sommer et al., 2015). The LxxIxE interaction interface that binds to BubR1 is completely conserved in all B56 isoforms (Wang J. et al., 2016), however, only a subset of these isoforms actually accumulate at the outer kinetochore (Nijenhuis et al., 2014). It is unclear why certain B56 isoforms bind preferentially to kinetochores, but this is an important issue to resolve, because it may reveal additional layers of PP2A-B56 regulation.

To experimentally address signaling specificity at the kinetochore, the ultimate goal should be to reconstitute kinetochores and use purified enzymes. Setting up such as system may seem like a mammoth task, but there has been some remarkable recent progress both in reconstituting the kinetochore *in vitro* from purified components and in isolating functional kinetochores from yeast (Akiyoshi and Biggins, 2012; Pesenti et al., 2016; Weir et al., 2016; Hinshaw and Harrison, 2018). The most straightforward approach, however, is to attempt to characterize the regulation in cells by using small molecule inhibitors or genetic manipulations to alter kinase or phosphatase activity. However, in this case, as discussed extensively above, care should be taken to rule out the inevitable indirect effects. I would therefore now like to finish by discussing some general experimental approaches that can be used to dissociate the direct from indirect effects of kinase or phosphatase inhibition *in vivo*.

## Indirect effects between kinase and phosphatase

**Figure 4:**
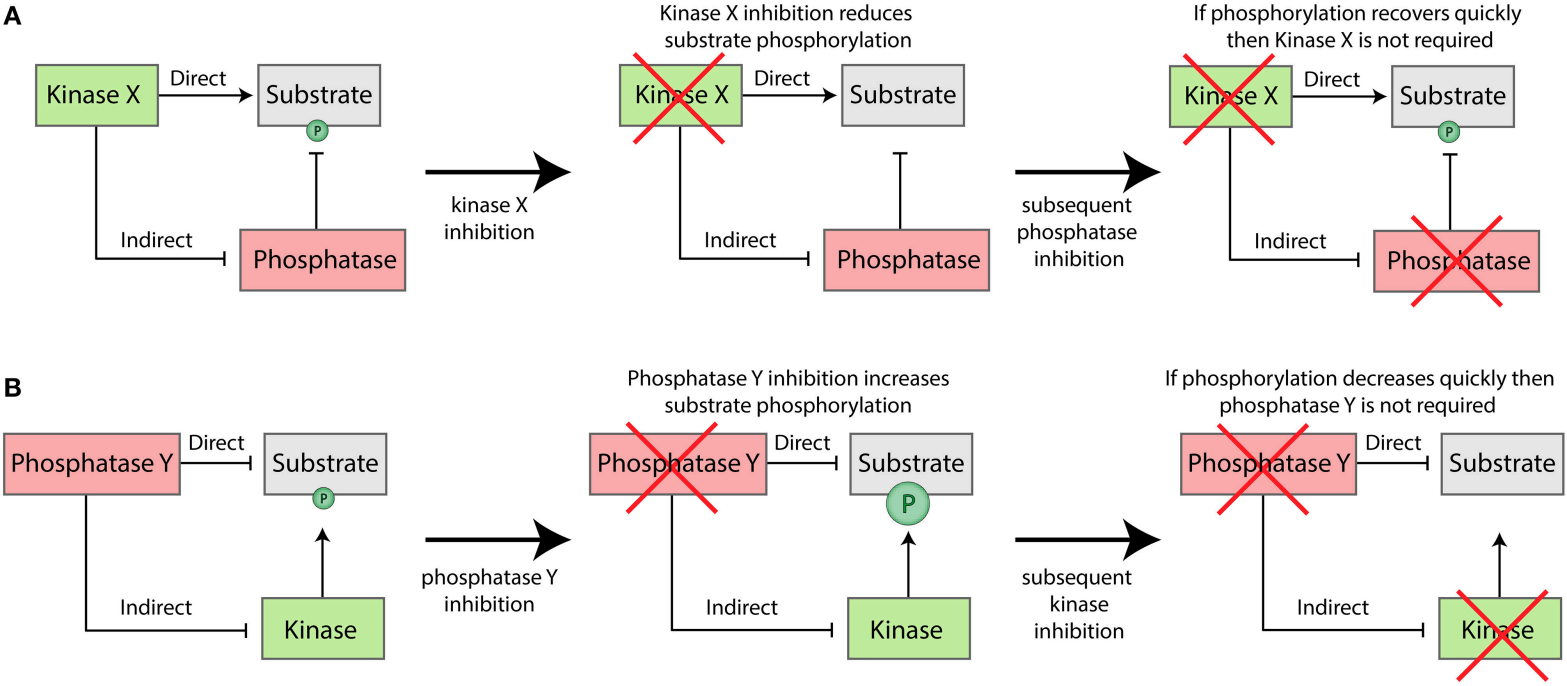
Generic approaches that can be used to dissociate the direct and indirect effects of kinase **(A)** or phosphatase **(B)** inhibition *in vivo*.

If kinase inhibition causes a target protein to become dephosphorylated, then this could be due to a decrease in the rate of phosphorylation, an increase in the rate of dephosphorylation, or both. A very simple way to discriminate between these two possibilities, is to first inhibit the kinase until the substrate becomes dephosphorylated and then subsequently inhibit the phosphatase (for example, with broad-spectrum PP1/PP2A inhibitors). If rapid re-phosphorylation is observed under these conditions, then this would imply that activity of the inhibited kinase is not required for this phosphorylation (see Figure 4A). The implication, therefore, is that kinase inhibition reduced phosphorylation by enhancing the rate of dephosphorylation. Similarly, if a specific phosphatase is inhibited and substrate phosphorylation increases, this could be either due to reduced dephosphorylation, enhanced phosphorylation, or both. In this case, if the respective kinase is known, then combined inhibition of this kinase can also be used to distinguish between these two possibilities (see Figure 4B).

Experiments such as these may help to determine whether PP1-Ska1 dephosphorylates the Knl1-MELTs directly or indirectly (via Mps1 inhibition), and whether Aurora B regulates Mps1 directly or indirectly (via PP1-Knl1 or PP1-Ska inhibition). When performing these experiments, it is important to measure the rates of (de)phosphorylation and to consider the fact that pharmacological inhibition is never absolute. Finally, when the indirect effects are known, it is possible to perform more elaborate experiments to determine their relative importance: for example, the downstream effects of Aurora B inhibition can be quantified in the context of Knl1 mutations that prevent activation of PP1-Knl1.

## Indirect effects between kinases

If kinase inhibition reduces target protein phosphorylation without increasing phosphatase activity, then this “direct” effect could still be mediated by another kinase. Often this can be ruled out by examining the kinase consensus motif and using *in vitro* kinase assays, however, there are still situations when it is difficult to distinguish between two kinases using this approach alone. For example, as discussed extensively above, Mps1 and Plk1 both phosphorylate the KMN network and have overlapping substrate specificities *in vitro*. Inhibiting these enzymes individually or in combination is one simple way to probe their relative contributions (Von Schubert et al., 2015; Ikeda and Tanaka, 2017). However, it is important to also consider that Mps1 indirectly controls Plk1 kinetochore levels by regulating Bub1 recruitment (Figure 1, arrow 15). Therefore, to validate whether the downstream effects of Mps1 inhibition are direct, it will be important to verify that substrate phosphorylation is not recovered if Plk1 activity is artificially rescued at kinetochores.

## Indirect effects between phosphatases

In general, determining specificity is more challenging for the phosphatases than it is for kinases, due to their relative weak substrate sequence preferences. That is not to say that there are no specificity determinants: PP2A-B55, for example, prefers to dephosphorylate phospho-threonine residues within motifs that are flanked by polybasic residues (Mccloy et al., 2015; Cundell et al., 2016; Godfrey et al., 2017; Hein et al., 2017). However, it has not been possible to define clear consensus motifs, at least for PP1 and PP2A, that would help to characterize their specific substrates *in vivo*. Therefore, care must be taken when implicating a phosphatase directly in substrate dephosphorylation. Even if removal of a specific phosphatase subcomplex prevents dephosphorylation without affecting kinase activity, this could still be mediated indirectly via a secondary phosphatase; as proposed previously for PP2A-B56 on KNL1-MELT phosphorylation (Nijenhuis et al., 2014). Once the molecular coupling between these phosphatases is known, however, then mutations can be used to rescue the downstream phosphatase and confirm whether the effects are direct or indirect. In this respect, double mutants to inhibit kinetochore PP2A-B56 whilst rescuing PP1-Knl1 could definitively test which phosphatase complex is crucial for SAC silencing.

## Concluding remarks

This review highlights how both kinetochore-microtubule attachments and the SAC are coregulated at the KMN network. It is important to stress that both of these processes can also be regulated from other areas of the kinetochore as well. This is exemplified by the outermost “fibrous corona” in human cells, which is an expanded region that forms around kinetochores during prometaphase to aid the capture of microtubules (Cassimeris et al., 1990; Thrower et al., 1996; Hoffman et al., 2001; Magidson et al., 2015; Wynne and Funabiki, 2015). Integral to the structure of the corona is the Rod/Zwilch/ZW10 (RZZ) complex, which forms oligomers that drive rapid corona expansion (Gama et al., 2017; Mosalaganti et al., 2017; Gassmann et al., 2018; Rodriguez-Rodriguez et al., 2018; Sacristan et al., 2018). The RZZ complex also helps to engage the SAC and many different SAC proteins localize to the expanded corona (Basto et al., 2000; Buffin et al., 2005; Kops et al., 2005; Silió et al., 2015; Wynne and Funabiki, 2015; Gassmann et al., 2018; Rodriguez-Rodriguez et al., 2018; Sacristan et al., 2018). In fact, the ability of the corona to support SAC signaling may help to explain the puzzling recent observations that Bub1 is dispensable for the SAC in human cells (Currie et al., 2018; Raaijmakers et al., 2018). It will be important to understand how the corona collaborates with the KMN network to regulate chromosome segregation, and in particular, how the enzymes at the KMN network may drive corona assembly and disassembly. Very recent work has begun to shed light on this interplay (Rodriguez-Rodriguez et al., 2018; Sacristan et al., 2018).

A critical feature of KMN regulation, that may be important for corona regulation too, is the interplay between kinases and phosphatases. Whilst it is very easy to adopt a kinase centric view of signaling, the danger is that this could lead to important regulatory inputs being missed or misinterpreted. A good example is the widespread use of FRET reporters to monitor “kinase” activity (Morris, 2013), which in truth, only ever readout the net effect of kinases and phosphatases that act on those reporters. If a specific FRET reporter detects changes in activity, then the frequent conclusion is that this reflects reciprocal changes in upstream kinase activity. However, unless this can be explained molecularly, and preferably rescued by reversing those molecular changes, then the reporter could equally be measuring changes in phosphatase activity. A good case in point is the Aurora B FRET reporter, which detects reduced activity at the outer kinetochore following biorientation and tension (Liu et al., 2009). Many theories have been put forward to explain tension-sensing, including inter/intra-kinetochore distance changes and structural changes within the kinetochore itself, but most of these models focus on the ability of tension to restrict Aurora B from accessing its outer kinetochore substrates. However, as pointed out recently by others (Lampson and Grishchuk, 2017), tension may also impact directly on local phosphatase activity (Vallardi et al., 2017). In fact, it may be difficult to discriminate between these two possibilities because Aurora B exhibits bistable activity in the presence of a phosphatase (Zaytsev et al., 2016); therefore, phosphatase activation may switch-off Aurora B activity. This bistable behavior could allow a steep gradient of Aurora B activity to form around kinetochores (Zaytsev et al., 2016), and alterations to kinase and/or phosphatase activities could potentially modulate this gradient to allow tension-sensing (Gelens et al., 2018). It will be important to characterize which phosphatases can deactivate Aurora B at the kinetochore, and how their activities may change upon kinetochore-microtubule attachment and tension.

The discussion about Aurora B nicely illustrates one important final point: the regulatory processes at the kinetochore could never be fully explained by considering either kinase or phosphatase inputs in isolation. These antagonistic enzymes work together in many different ways to define a signaling response (Gelens et al., 2018). At the KMN network, they work together within a large network that includes multiple different enzymes, which are interconnected in a way that can produce complex biological outputs. Although reductionist biology has provided much of this regulatory framework, these key biological outputs are only ever likely to be truly explained by shifting toward more holistic approaches that can make sense out of such complexity. Hopefully, by collating information from a wide variety of labs into a single model of KMN network regulation, this article may help progress toward this ultimate goal.

## Author contributions

The author confirms being the sole contributor of this work and approved it for publication.

### Conflict of interest statement

The author declares that the research was conducted in the absence of any commercial or financial relationships that could be construed as a potential conflict of interest.
